# NF-κBp50 and HDAC1 Interaction Is Implicated in the Host Tolerance to Infection Mediated by the Bacterial Quorum Sensing Signal 2-Aminoacetophenone

**DOI:** 10.3389/fmicb.2017.01211

**Published:** 2017-06-30

**Authors:** Arunava Bandyopadhaya, Amy Tsurumi, Laurence G. Rahme

**Affiliations:** ^1^Department of Surgery, Massachusetts General Hospital and Harvard Medical School, BostonMA, United States; ^2^Department of Microbiology and Immunobiology, Harvard Medical School, BostonMA, United States; ^3^Shriners Hospitals for Children Boston, BostonMA, United States

**Keywords:** quorum sensing, 2-aminoacetophenone, inflammatory cytokines, NF-κB, CBP/p300, HDAC1

## Abstract

Some bacterial quorum sensing (QS) small molecules are important mediators of inter-kingdom signaling and impact host immunity. The QS regulated small volatile molecule 2-aminoacetophenone (2-AA), which has been proposed as a biomarker of *Pseudomonas aeruginosa* colonization in chronically infected human tissues, is critically involved in “host tolerance training” that involves a distinct molecular mechanism of host chromatin regulation through histone deacetylase (HDAC)1. 2-AA’s epigenetic reprogramming action enables host tolerance to high bacterial burden and permits long-term presence of *P. aeruginosa* without compromising host survival. Here, to further elucidate the molecular mechanisms of 2-AA-mediated host tolerance/resilience we investigated the connection between histone acetylation status and nuclear factor (NF)-κB signaling components that together coordinate 2-AA-mediated control of transcriptional activity. We found increased NF-κBp65 acetylation levels in 2-AA stimulated cells that are preceded by association of CBP/p300 and increased histone acetyltransferase activity. In contrast, in 2-AA-tolerized cells the protein–protein interaction between p65 and CBP/p300 is disrupted and conversely, the interaction between p50 and co-repressor HDAC1 is enhanced, leading to repression of the pro-inflammatory response. These results highlight how a bacterial QS signaling molecule can establish a link between intracellular signaling and epigenetic reprogramming of pro-inflammatory mediators that may contribute to host tolerance training. These new insights might contribute to the development of novel therapeutic interventions against bacterial infections.

## Introduction

Bacterial quorum sensing (QS) systems regulate the expression of multiple virulence factors via small excreted signaling molecules that play important roles in bacterial cell-to-cell communication and inter-kingdom interaction that favor infection (Ng and Bassler, 2009; Parker and Sperandio, 2009; Rutherford and Bassler, 2012; Starkey et al., 2014; Maura et al., 2016). The QS systems of the ESKAPE pathogen *Pseudomonas aeruginosa* are the most well studied systems and shown to be required for acute and chronic infections (Kerr and Snelling, 2009; Gellatly and Hancock, 2013). *P. aeruginosa* regulates many of its virulence functions via the QS systems, LasR, RhlR, and MvfR (pqsR; Jimenez et al., 2012). LasR and RhlR rely on the signaling molecules N-acyl-homoserine lactones (AHLs; Jimenez et al., 2012), while the quinolone-dependent QS system, MvfR (PqsR) relies on the 4-hydroxy-2-alkylquinolines (HAQs) signaling molecules, 2-heptyl-3,4-dihydroxyquinoline (PQS, Pseudomonas Quinolone Signal), and HHQ (4-hydroxy-2-heptylquinoline) (Deziel et al., 2005; Diggle et al., 2006; Xiao et al., 2006; Williams and Camara, 2009; Drees and Fetzner, 2015; Drees et al., 2016). Apart from their role as QS signal molecules, AHLs and HAQs also modulate immune responses, promote apoptosis, and control chemotaxis, cell proliferation and phagocytosis by regulating host intracellular signaling pathways (Kravchenko et al., 2008; Rumbaugh and Kaufmann, 2012; Holm and Vikstrom, 2014).

Recently, we have reported that MvfR in addition to HAQs also controls the synthesis of the non-HAQ molecule 2-aminoacetophenone (2-AA; Kesarwani et al., 2011; Bandyopadhaya et al., 2012; Que et al., 2013), which is abundantly produced in *P. aeruginosa*-infected human tissues (Cox and Parker, 1979; Scott-Thomas et al., 2010). 2-AA modulates both bacterial and host functions (Kesarwani et al., 2011; Bandyopadhaya et al., 2012; Que et al., 2013). In the bacterium, 2-AA silences the acute virulence functions of the MvfR QS system (Kesarwani et al., 2011) by binding and inhibiting the enzymatic activity of PqsBC (Drees et al., 2016) and promotes antibiotic tolerance at least by interfering with the bacterial translation apparatus (Que et al., 2013). In the host, it acts as an inter-kingdom immunomodulatory signal (Bandyopadhaya et al., 2012) and promotes metabolic changes (Tzika et al., 2013; Bandyopadhaya et al., 2016a). It dramatically reduces *P. aeruginosa* induced mortality by limiting pathogen-induced inflammation and tissue damage (Bandyopadhaya et al., 2012), while enables *P. aeruginosa* to persist at a high burden level (Bandyopadhaya et al., 2012). Its impact on host metabolism may also favor chronic infection (Tzika et al., 2013; Bandyopadhaya et al., 2016a). More recently, we have uncovered that 2-AA acts as a critical mediator (training agent) of host tolerance/resilience (HT/R) against *P. aeruginosa* through a distinct molecular mechanism of host chromatin regulation that involves histone deacetylases (HDAC)1 expression and activity (Bandyopadhaya et al., 2016b). HT/R to infections is defined as the host’s ability to limit pathogen triggered damage, while permitting pathogen persistence (Raberg, 2014; Richardson, 2016; Meunier et al., 2017; Soares et al., 2017). The host copes with a pathogenic encounter without a reduction in fitness (Ayres and Schneider, 2012; Medzhitov et al., 2012; Soares et al., 2014) and avoids harmful inflammatory responses that can occur during immune-driven resistance (Schmid-Hempel, 2009). Our understanding of the biological mechanisms mediating mutual pathogen–host adaptation and the causes and consequences of variation in HT/R is extremely limited.

Innate immune mechanisms relay on the recognition of conserved patterns of pathogens, through pattern recognition receptors that activate intracellular signaling pathways ultimately regulating the expression of pro-inflammatory mediators, which together coordinate the early host response to infection, that is required for the activation of adaptive immunity (Mogensen, 2009). The magnitude and duration of the inflammatory response is tightly regulated by endogenous host signaling molecules in order to avoid the self-damaging immunopathology that results from an uncontrolled inflammatory response (Iwasaki and Medzhitov, 2004; Newton and Dixit, 2012). On the other hand, pathogens respond to the threat imposed by the immune system by adopting a series of strategies that aim at escaping or reducing the effectiveness of the host defense mechanisms by interfering with molecules involved in inflammatory signaling, eventually promoting long-standing association with host and persistent infections (Ruby and Monack, 2011; Reddick and Alto, 2014). However, the biological mechanisms by which pathogens act upon hosts to persist and support their survival remains largely elusive.

**FIGURE 1:**
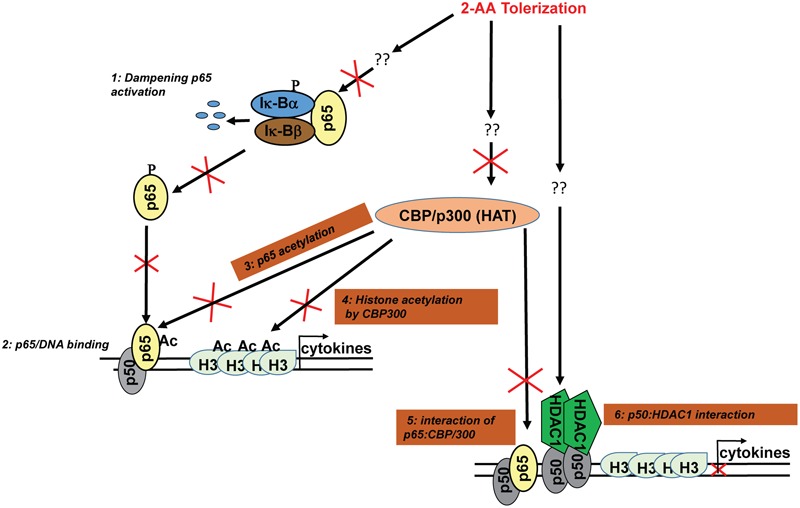
2-AA-mediated immunomodulation. Our studies have shown that 2-AA tolerization dampens the activation of NF-κB pathway (1) (Bandyopadhaya et al., 2012). The DNA binding activity of NF-κB p65 (2) (Bandyopadhaya et al., 2012) is impaired due to reduced acetylation level of p65 in 2-AA-tolerized cells (3 this study). 2-AA tolerization reduces histone acetylation (H3) via downregulation of CBP/p300 HAT activity and expression (4 this study) and the protein–protein interaction between p65 and CBP/p300 (5 this study). The upregulation of HDAC1 initiates HDAC1–p50 interaction and p50 homodimer–HDAC1 complexes are recruited at targeted sites (6 this study). Consequently, HDAC1 acts to deacetylate H3 and dampens transcription of cytokines.

The intracellular host milieu is hostile for bacterial cells because of the host dynamic defense system, which is primarily controlled by the innate effector cells. The nuclear factor (NF)-κB signaling has an essential role in inflammation and innate immunity (Bhatt and Ghosh, 2014). The prototypical NF-κB is a heterodimer consisting of two subunits, p65 and p50. The p65 has the strongest transcriptional activity, whereas the p50 subunit lacks transactivation domain and accounts for the strong DNA-binding affinity (Gilmore, 2006; Sharma et al., 2015). We have reported that the degradation and dissociation of Iκ-Bα from NF-κB, the NF-κBp65 translocation to the nucleus and binding to the DNA are impaired during 2-AA-mediated tolerance (Bandyopadhaya et al., 2012; **Figure 1**).

NF-κB can influence transcription by recruitment of co-activators such as the acetyltransferase CREB (c-AMP response element binding) binding protein (CBP)/p300 (Vo and Goodman, 2001). The histone acetyltransferase (HAT) domain of CBP/p300 modulates transcription by catalyzing the acetylation of promoter-bound histones (Yuan et al., 2013). In addition, HATs also regulate gene expression through acetylation of NF-κB p65, which subsequently determines their nuclear interactions with DNA and other transcriptional regulators (Yang et al., 2010). This acetylation is balanced by the removal of acetyl groups by HDACs, which are associated with chromatin compaction and transcriptional repression (Delcuve et al., 2012). Bacteria can impede the dynamic regulation of HATs and HDACs which may affect host innate immunity (Hamon and Cossart, 2008; Bierne et al., 2012).

Here we investigate 2-AAs’ regulation on host intracellular signaling and the effects of this regulation in 2-AA-mediated immunomodulation. Our results provide additional new insights into the 2-AA-mediated mechanism of tolerization that might contribute to therapeutic intervention strategies against bacterial infections.

## Materials and Methods

### Cell Culture

THP-1 Blue cells (human monocytic leukemia line with an NF-κB-inducible reporter; Invivogen, United States) and RAW264.7 cells carrying the NF-κB luciferase plasmid (IMGENEX, United States) were maintained in RPMI 1640 medium (Life Technologies, United States) and Iscove’s modified Dulbecco’s medium (IMDM, Gibco), respectively. All media was supplemented with 10% heat-inactivated FBS/1% Antibiotic-Antimycotic/2 mM L-glutamine/10 mM HEPES (all from Gibco, United States). The cells were cultured in T-75 flasks (Falcon, United States) and used between passages 2 and 3. HDAC1 knock down and vector control stable cell lines were maintained in complete IMDM (Gibco) with puromycin (Bandyopadhaya et al., 2016b). Cells were maintained in a humidified incubator at 37°C in 5% CO_2_.

### 2-AA Tolerization Assay

**FIGURE 2:**
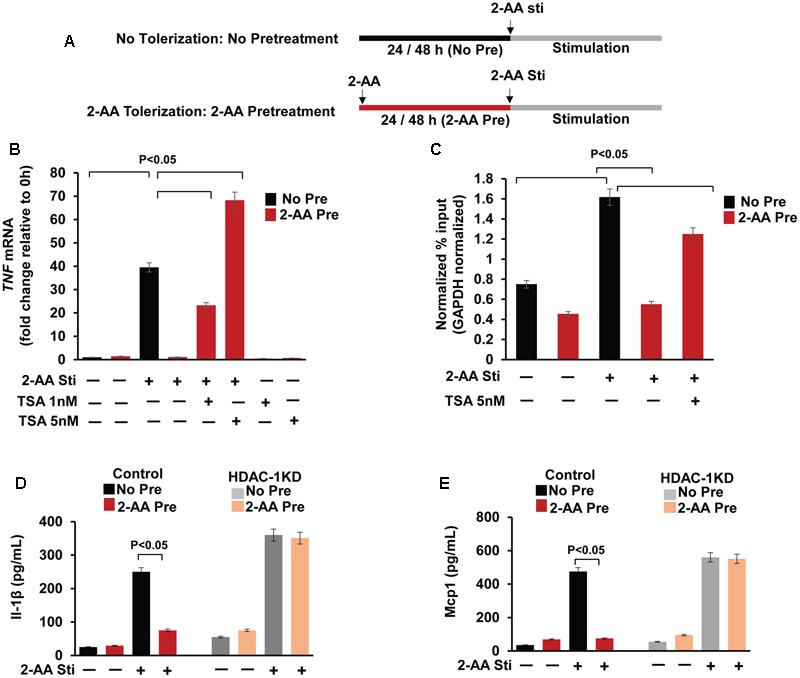
Pharmacological and genetic inhibition of HDAC1 rescue the 2-AA-mediated immunomodulation. **(A)** Schematic representation of the 2-AA treatments. The human monocytes THP-1 cells and mouse macrophage RAW264.7 cell cells were left untreated (No Pre: No Tolerization) or pretreated with 2-AA for 24 h or 48 h (2-AA Pre: 2-AA tolerization) respectively, and then stimulated (Sti) with 2-AA. **(B)** Expression of *TNF* (1 h) was measured in non-pretreated, 2-AA pretreated and 2-AA + TSA pretreated THP-1 cells following 2-AA stimulation. Transcript levels were assessed by qRT-PCR and normalized to *GAPDH*. **(C)** ChIP assay of H3K18ac at the *TNF* promoter of 2-AA-pretreated, 2-AA and TSA pretreated or non-pretreated THP-1 cells following 3 h 2-AA stimulation, assessed by qRT-PCR with primer covering the promoter site region of *TNF* relative to *GAPDH.* **(D,E)** ELISA of IL-1β and Mcp1 secretion in culture supernatant of 2-AA pretreated vector control RAW264.7 and HDAC1 KD cells following 6 h 2-AA stimulation (*n* = 3; means ± SDs; *p* < 0.05, Student’s *t*-test).

THP-1 cells (10^5^/mL) were plated in 24-well plates. Cells in the treatment groups were pretreated with 400 μM 2-AA (Sigma-Aldrich, United States) for 24 h; pretreated and non-pretreated cells were washed with 1× phosphate-buffered saline (PBS) and kept in fresh medium (**Figure 2A**). Cells were stimulated with 200 μM 2-AA for the durations indicated in the figures. Similarly, murine macrophage RAW264.7 cells were pretreated (or not) with 800 μM 2-AA for 48 h, then stimulated with 400 μM 2-AA.

### RNA Isolation and Quantitative RT-PCR

Total RNA was isolated from approximately 1.2 × 10^6^ cells with the RNeasy Mini Kit (Qiagen, United States) and cDNA was prepared with the RETROscript^TM^ Kit (Ambion^®^ Life Technologies, United States), as per the manufacturer’s instruction. Real-time PCR was conducted using the Brilliant II SYBR green super mix (Agilent, United States) and primer sets for human tumor necrosis factor (*TNF*; forward: AACATCCAACCTTCCCAAACG, reverse: CTCTTAAACCCCCGAATCCCAG) and *GAPDH* (forward: CAACAGCGACACCCACTCCT, reverse: CACCCTGTTGCTGTAGCCAAA). Expression of cytokines was normalized to *GAPDH* with the ΔCt method and relative expression was calculated relative to non-pretreated and unstimulated control cells. The assay was conducted in triplicate; means and standard deviations were calculated for each group.

### Western Blot

Whole cell lysates were prepared in RIPA buffer (Cell Signaling Technology, United States) supplemented with protease inhibitor cocktail (Sigma). The concentration of protein was determined from each sample using the Bradford protein assays kit (Bio-Rad Laboratories, Hercules, United States). Fifteen to twenty-five micrograms of total protein was added to 1× Laemmli buffer, boiled for 10 min, separated by SDS 7.5–15% polyacrylamide gel electrophoresis (PAGE) in 25 mM Tris/250 mM glycine/0.1% SDS buffer (Bio-Rad), and transferred to PVDF membranes (Bio-Rad). The membranes were blocked for 2 h in 5% non-fat milk. The membranes were then washed three times in TBS-T (20 mM Tris–HCl/150 mM NaCl/0.1% Tween 20) and probed overnight with antibodies specific for acetyl-CBP/p300K1499, acetyl-NF-κBp65K310, HDAC1 (Cell Signaling Technology), CBP, p300, p65, and p50 (Santa Cruz Biotechnology, United States) at a dilution of 1:1,000, and mouse anti-β-actin (Santa Cruz Biotechnology) at a dilution of 1:2,000. Following three washes in TBST, the membranes were incubated with secondary horseradish peroxidase (HRP)-conjugated antibody: goat anti-rabbit (Santa Cruz Biotechnology, United States) or goat anti-mouse secondary antibodies (Promega, United States). The membranes were washed three times in TBST and then developed by SuperSignal West Pico Chemiluminescent Substrate (Thermo Scientific, United States), as per the manufacturer’s instructions. Protein bands were quantified by ImageJ software^1^.

### Preparation of Nuclear Fraction

Nuclear fractions were extracted from cells with a Nuclear Extract kit (Active Motif, Carlsbad, United States) as per manufacturer’s instructions. Briefly, cells were scraped into a hypotonic buffer supplemented with phosphatase inhibitors and kept on ice. The cell suspension was centrifuged at 12,000 rpm for 15 min at 4°C. The cell pellet was suspended in nuclear lysis buffer, rocked lightly for 30 min at 4°C, and centrifuged at 12,000 rpm for 15 min at 4°C. Nuclear protein was quantified by Bradford protein assay.

### Measurement of HAT Activity

HAT activity was performed by using a non-radioactive HAT activity assay kit (Active Motif, United States), as per manufacturer’s instructions. Briefly, the nuclear protein (3 μg) was incubated in HAT assay buffer containing mixture of acetyl-CoA and histone H3 peptide for 30 min at RT. The reaction was terminated by stop solution. The sample was incubated with developing solution for 15 min at RT. Fluorescence (excitation, 380 nm; emission, 460 nm) was measured using a microplate reader (Tecan Group Ltd, Switzerland). HAT activity was expressed as arbitrary fluorescence units (AFU).

### Histone H3 Modification Array

The binding of a 2-AA treated nuclear fraction was used to identify the substrates of histone modifying enzymes using Pre-Sure Histone H3 peptide Array ELISA kit (EpiGentek) according to the manufactures protocol. Briefly, 50 μL of 2-AA-treated nuclear fraction was added to the histone H3K acetylated peptide-coated well and incubated for 1 h. Following three washes developer solution was added for 10 min at RT and the absorbance at 450 nm was determined using a Sunrise plate reader.

### Chromatin Immunoprecipitation

Cells were cross-linked in 1% methanol-free formaldehyde for 10 min, then placed in 0.125 M glycine for 5 min at RT. Using the *tru*ChIP^TM^ High Cell Chromatin Shearing kit (Covaris, United States), cells were prepared for sonication according to the manufacturer’s protocol. Approximately 1 × 10^7^ cells were places in a 12 mm × 12 mm tube and subjected to shearing with the Covaris S220 sonicator for 8 min (140 peak power, 5 duty factor, 200 cycles/burst). The Magna ChIP^TM^ A/G Kit (Millipore, United States) was used for the subsequent immunoprecipitations (IPs) according to the manufacturer’s protocol. Briefly, chromatin from approximately 10^6^ cells was incubated overnight at 4°C with 4 μg of anti-acetyl H3K18 (Abcam, United States) chromatin IP (ChIP)-grade antibody and 20 μL of A/G magnetic beads. The beads were washed serially (5 min each) with low-salt wash buffer, high-salt wash buffer, LiCl wash buffer, and TE buffer from the kit at 4°C. Chromatin was eluted with elution buffer containing Proteinase K at 62°C for 4 h, then incubated at 95°C for 10 min. DNA was isolated by column purification. Real-time PCR was performed with the Brilliant II SYBR green super mix (Agilent, United States) and primer sets to amplify various regions of *TNF* (forward: CCCCCTCGGAATCGGA, reverse: GAGCTCATCTGGAGGAAGCG) and *GAPDH* (forward: CGGTGCGTGCCCAGTT, reverse: CCCTACTTTCTCCCCGCTTT) loci. Normalized values were calculated by the percent-input method relative to the *GAPDH* promoter locus. The assay was conducted in triplicate; means are reported with standard deviations.

### Co-immunoprecipitation

For protein–protein interaction assays, whole cell lysates were extracted in 1× TNT buffer [20 mM Tris–HCl, pH 7.5/200 mM NaCl/Triton X-100/0.1 M phosphatase inhibitor cocktails 1 and 2 (ingredients from Sigma-Aldrich)]. A total of 100 μg aliquots of whole cell extracts were subjected to IP in 0.1× TNT buffer. IP was performed with 2 μg of HDAC1, p300, CBP, p50, or p65 (Santa Cruz Biotechnology, Santa Cruz, CA, United States) and 50 μL of Pierce protein A/G agarose beads (Thermo Scientific, United States)at 4°C for 3 h. After washing three times with 0.1× TNT buffer, the bound proteins eluted in the Laemmli loading buffer supplemented with 100 mM dithiothreitol by boiling for 10 min, the precipitated proteins were analyzed by Western blotting, as described above.

### Measurement of IL-1β and Mcp1 by Enzyme-Linked Immunosorbent Assay

Murine interleukin (IL)-1β and monocyte chemotactic protein (Mcp)1 were measured by ELISA using the Quantikine kits (R&D Systems, MN, United States), following manufacturer’s instructions. Briefly, culture supernatants were added to antibody-coated ELISA plates and kept at RT for 2 h. Following four washes, HRP-conjugated streptavidin/biotinylated antibody solution was added to plates and kept at RT for 2 h. The assay was developed with the tetramethylbenzidine substrate reagent and incubated at RT for 20 min. The absorbance was assessed at 450 nm using a Sunrise plate reader.

### MTT Assay for Cell Cytotoxicity

The cytotoxicity of cells treated with C 646 (Abcam, United States) was measured by 3-(4,5-dimethyl-2-thiazolyl)-2,5-diphenyl-2*H*-tetrazolium bromide (MTT) assay. MTT (Sigma) stock solution (5 mg/mL PBS) was further diluted 1:5 in PBS. Cells were exposed to 200 μL of this working solution in 96-well culture plates where dissolved MTT was allowed to convert to insoluble purple formazan via mitochondrial activity for 2 h at 37°C in humidified incubator containing 5% CO_2._ The medium was discarded, formazan was solubilized with 95% isopropanol–5% formic acid for 10 min and absorbance was measured at 555 nm (reference wavelength 690 nm) using a Sunrise plate reader. The concentration of C646 used did not induce cytotoxicity (**Supplementary Figure S1**).

### Pharmacological Inhibitors

For treatment, THP-1 cells were incubated with HDAC1 inhibitor TSA (trichostatin A; Villagra et al., 2010) (1 or 5 nM, Sigma-Aldrich) and CBP/p300 inhibitor C646 (0.1 μM; Zhao et al., 2015).

### Statistical Analysis

For the analysis of statistical significance, the data were analyzed using the Student’s *t*-test and *P*-value <0.05 was considered significant for all experiment.

## Results

### HDAC1 Controls the Dampening of Pro-inflammatory Responses in 2-AA-Tolerized Cells

HDACs mediate histone deacetylation and thus suppress gene transcription (Delcuve et al., 2012). Our previous study demonstrates that HDAC1 regulates the deacetylation of H3K18 at the *TNF-α* promoter and TNF secretion in 2-AA pretreated (tolerized) cells (Bandyopadhaya et al., 2016b; **Figure 1**). We interrogated reversion of the deacetylation effect promoted by 2-AA by assessing the *TNF* mRNA levels in 2-AA-tolerized THP-1 cells in the presence of Class I/II HDAC inhibitor TSA (**Figure 2A**). Indeed, **Figure 2B** shows that in presence of TSA there is a significant increase of *TNF* mRNA expression in 2-AA pretreated cells. We additionally analyzed H3K18 acetylation at the *TNF* promoter. Pretreatment with 2-AA and TSA enriched H3K18 acetylation at the *TNF* promoter compared to 2-AA-tolerized THP-1 cells (**Figure 2C**). Furthermore, **Figures 2D,E** show that the secretion of the other major pro-inflammatory cytokines IL-1β and Mcp1 is significantly dampened in 2-AA-tolerized cells, whereas depletion of HDAC1 counteracts the IL-1β and Mcp1 secretion in 2-AA-tolerized cells.

### 2-AA-Tolerized Cells Lack NF-κB p65 Acetylation

**FIGURE 3:**
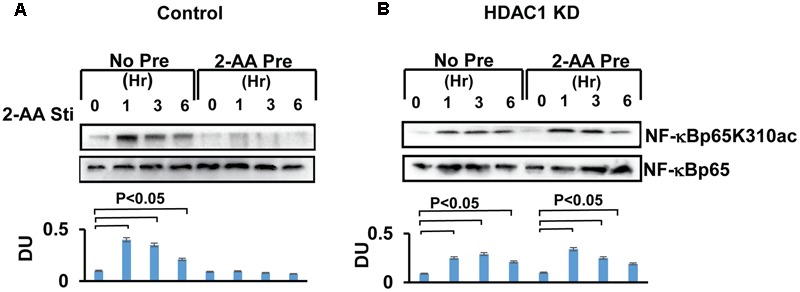
2-AA tolerization decreases the acetylation of NF-κBp65. Immunoblot showing decreased NF-κBp65K310 acetylation in 2-AA-tolerized RAW264.7 cells **(A)**. HDAC1 KD restored NF-κBp65K310 acetylation in 2-AA-tolerized cells following 2-AA stimulation **(B**). Data are representative of three independent experiments. An asterisk (^∗^) indicates significant difference from untreated cells (*P* < 0.05). DU, densitometry units, bars represent SD.

Post-translational modifications, particularly acetylation of NF-κB p65 enhances the DNA-binding activity of p65 at the promoter of pro-inflammatory cytokines regulated by NF-κB (Chen et al., 2005; Bhatt and Ghosh, 2014). Our prior study demonstrated that 2-AA tolerization dampens the DNA-binding activity of p65 (Bandyopadhaya et al., 2012; **Figure 1**). Therefore, we sought to determine changes in the level of p65 acetylation following 2-AA treatment by using wild type and knockdown HDAC1 RAW264.7 cells. **Figure 3** shows that although p65 acetylation is promoted following 2-AA stimulation, it is absent in 2-AA-tolerized cells (**Figures 1, 3A**). On the other hand, knockdown of HDAC1 restores p65 acetylation in 2-AA-tolerized cells (**Figure 3B**).

### 2-AA Induces H3K18 Acetylation by CBP/p300

**FIGURE 4:**
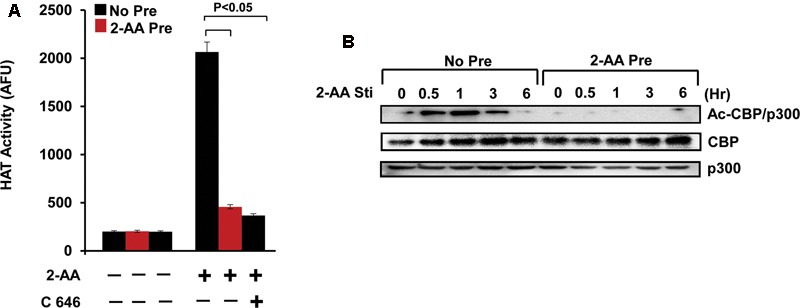
2-AA modulates CBP/p300-mediated HAT activity and the acetylation of CBP/p300. **(A)** HAT activity in nuclear lysate of 2-AA stimulated and 2-AA-tolerized THP-1 cells following 1 h 2-AA stimulation +/– of C646 (*n* = 3; means ± SDs; *p* < 0.05, Student’s *t*-test). **(B)** Immunoblot showing decreased CBP/p300 acetylation in 2-AA-tolerized THP-1 cells. Data are representative of three independent experiments.

**FIGURE 5:**
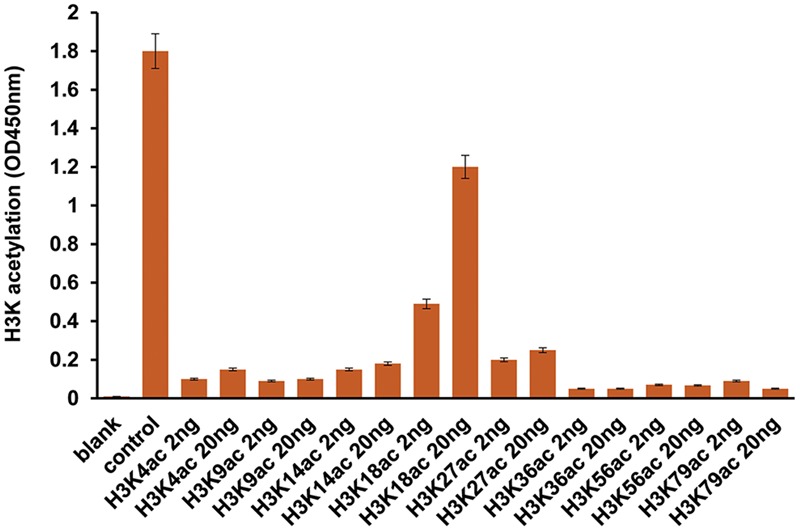
2-AA modulates H3K18 acetylation. Screening for Histone H3 acetylation following 2-AA stimulation. Nuclear extraction was prepared from THP-1 cells 1 h post-stimulation with 2-AA. The interaction of the enzyme in the sample solution with the acetylated H3K peptides was measured by using Histone H3 Peptide Array ELISA kit (number of replicates = 2; means ± SDs).

Previously, we have found that 2-AA induces HAT activity and this induction was reduced in 2-AA-tolerized cells (Bandyopadhaya et al., 2016b). Our present data demonstrate that 2-AA-mediated HAT activity is regulated specifically by CBP/p300, since C646 (the specific inhibitor of p300/CBP HAT activity) abrogated nuclear HAT activity in THP-1 cells stimulated with 2-AA (**Figure 4A**). 2-AA initially stimulates the acetylation of CBP/p300 at Lys1499, whereas 2-AA tolerization significantly dampens this acetylation mark in both THP-1 and RAW264.7 cells (**Figure 4B** and **Supplementary Figures S1, S2**). Therefore, we used the 2-AA stimulated nuclear fraction to screen various residues for H3K acetylation. **Figure 5** shows that 2-AA stimulated nuclear fractions have strong affinity toward K3K18 acetylated peptide, demonstrating that 2-AA modulates H3K18 acetylation, presumably by regulating the acetylation of CBP/p300 (**Figure 5**).

### 2-AA Enhances the Interaction between p50 and HDAC1 by Inhibiting p65 Phosphorylation and Disrupting p65 and CBP/p300 Interaction

**FIGURE 6:**
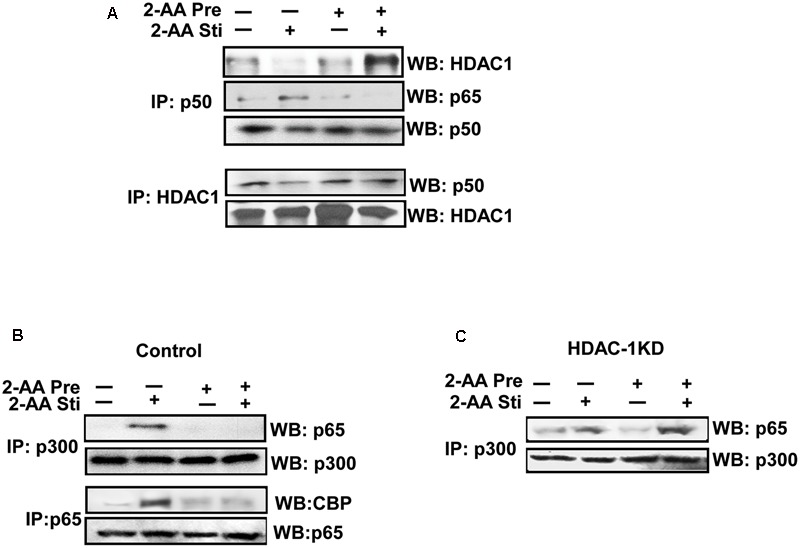
2-AA tolerization induces interaction between NF-κB p50 and HDAC1 and disrupts p65-CBP/p300 binding. **(A)** Co-IP assay showing p50/HDAC1 interaction in 2-AA stimulated and 2-AA-tolerized THP-1 cells following at 1 h 2-AA stimulation (top blot, far right band). **(B,C)**. Co-IP assay confirming that 2-AA tolerization inhibits the interaction of p65 with CBP and p300 in vector control RAW264.7 cells **(B)**, and demonstrating an increased interaction of p300-p65 in HDAC1 KD cells **(C)** following 1 h 2-AA stimulation (1 h). WB, Western blot; Co-IP, co-immunoprecipitation. Data are representative of three independent experiments.

NF-κB signaling enhances the interaction between the active p65–p50 heterodimer and CBP/p300, which displaces the silencing p50–p50 homodimer–HDAC1 complex from DNA (Zhong et al., 2002). We therefore investigated the protein–protein interactions mediated by 2-AA and observed that the upregulation of HDAC1 protein expression in 2-AA-tolerized cells (Bandyopadhaya et al., 2016b) was accompanied by enhanced interaction with p50 in THP-1 and RAW264.7 cell lines (**Figure 6A** and **Supplementary Figure 3a**) and disrupted interaction between p65 and CBP/p300 (**Figure 6B** and **Supplementary Figure 3b**). 2-AA cell tolerization prior to stimulation resulted in reduced CBP/p300 acetylation (**Figure 4B** and **Supplementary Figure S2**), a modification that dictates its binding partner (Zhong et al., 2002). Acetylation of lys310 of the p65 subunit enhances CBP/p300 HAT binding and disrupts HDAC1 binding to regulate transcriptional activity (Quivy and Van Lint, 2004) and this post-translational modification was also decreased in 2-AA-tolerized RAW264.7 cells, compared to non-tolerized cells that were stimulated only (**Figure 6B**). Importantly, **Figure 6C** shows that knockdown of HDAC1 restored p300 and p65 interactions in 2-AA-tolerized and re-exposed RAW264.7 cells that were otherwise disrupted in similar control cells. This shift in protein–protein interaction and HDAC1 regulation likely mediates changes in histone acetylation by 2-AA resulting in the attenuation of pro-inflammatory cytokine expression.

## Discussion

Pathogens may modulate host immune responses in a way to avoid clearance and potentially favor their long-term presence in the host tissues (Ruby and Monack, 2011; Reddick and Alto, 2014; Ribet and Cossart, 2015). Recently, we have demonstrated that the bacterial excreted QS small molecule 2-AA trains the host to tolerate a sustained presence of *P. aeruginosa* by a HDAC1-mediated epigenetic reprogramming (Bandyopadhaya et al., 2016b). In this study, we undertook further molecular analyses of alterations in intracellular signaling components and epigenetic regulators following 2-AA-mediated tolerization. Our results reveal that the 2-AA promoted host tolerance implicates NF-κBp50 and HDAC1 interaction. Several data are in support of this conclusion, including that: (i) 2-AA-tolerized cells lack NF-κBp65 acetylation; (ii) 2-AA modulates H3K18 acetylation via downregulation of CBP/p300 HAT activity and expression; (iii) 2-AA tolerization induces enhanced protein–protein interaction between NF-κBp50 and HDAC1 and reduces binding of CBP/p300 to NF-κB p65; (iv) HDAC1 knockdown restores acetylation of NF-κB p65 in 2-AA-tolerized cells; and (v) the pharmacological and genetic inhibition of HDAC1 rescue 2-AA-mediated immunomodulation observed. These findings provide additional details on the components and mechanism involved in the epigenetic reprogramming reported previously (Bandyopadhaya et al., 2016b) and strongly suggest that 2-AA tolerization triggers modulation of intracellular molecules that contributes to the shifts in innate immune cells from transcriptionally permissive to a repressive chromatin state that in turn augments host tolerance to pathogen’s long-term presence we reported previously (Bandyopadhaya et al., 2012, 2016b).

Previous studies have demonstrated that diminished NF-κB signaling (Medvedev et al., 2000; Dobrovolskaia et al., 2003; Bandyopadhaya et al., 2016b), p50–p50 homodimers (Ziegler-Heitbrock et al., 1994; Bandyopadhaya et al., 2012), chromatin remodeling (Bandyopadhaya et al., 2016b), and dampened inflammatory response (Biswas and Lopez-Collazo, 2009; Bandyopadhaya et al., 2012) have all been implicated in the induction of the tolerant state. NF-κB p50–HDAC1 complex represses transcription of major proinflammatory mediators, such as, *Tnf, IL-6*, and *cyclooxygenase-2* (*Cox-2*) (Oakley et al., 2005; Elsharkawy et al., 2010). Our data showed that HDAC1 is associated with deacetylation of H3K18 at *Tnf* promoters in the 2-AA-tolerized cells (Bandyopadhaya et al., 2016b). Here we provide evidence suggesting that inhibition of HDAC1 activity results in an increase of pro-inflammatory mediators in 2-AA-tolerized cells. We speculate that the increased interaction of p50 and HDAC1 in 2-AA-tolerized cells may orchestrate the transcriptional repression of pro-inflammatory genes. NF-κB p50-HDAC3-nuclear hormone receptor co-repressor interaction was found to be required to repress inflammatory activation in LPS-tolerized cells (Yan et al., 2012) that is different from the 2-AA-mediated tolerization (Bandyopadhaya et al., 2016b). Thus, HDAC1 and HDAC3 appear to be critically involved in immunomodulation by epigenetic reprogramming mediated by bacterial products, while HDAC1 also regulates the ability to tolerate pathogen burden (Bandyopadhaya et al., 2016b).

HDAC1 and NF-κB signaling coordinate to repress inflammatory genes during infection (Zhou et al., 2013). NF-κB influences transcription by recruiting p300/CBP, which can specifically acetylate the H3K18 residue (Jin et al., 2011) and simultaneously regulate p65 acetylation, leading to transcription (Quivy and Van Lint, 2004). It has been reported that of NF-κB acetylation is required for NF-κB-dependent transcription (Chen et al., 2005) and is regulated by the HDAC family of proteins, including HDAC1 (Ashburner et al., 2001). Here our results show that 2-AA tolerization promotes the interaction between p50 and HDAC1, with a coordinate loss of p65 (Lysine 310) acetylation and CBP/p300 binding with p65, which leads to a transcriptionally repressive state. It has been reported that Sirtuin 1 (SIRT1, Class III HDAC) deacetylates p65 at Lysine 310 to repress the pro-inflammatory responses during initiation of endotoxin tolerance (Liu et al., 2011). This study provides further demonstration of the intimate link between HDACs and NF-κB pathway, specifically between HDAC1 and components of the NF-κB signaling cascade in 2-AA-mediated host tolerance.

Based on our published and present results, we propose a model in **Figure 1**, in which 2-AA tolerization, which involves pretreatment and stimulation (**Figure 2A**), decreases CBP/p300 HAT activity, inhibits p65 and H3 acetylation, prevents p65 and CBP/p300 interaction, and in turn promotes p50–HDAC1 interaction, leading to a sustained 2-AA-mediated histone deacetylation, concomitant loss of active transcription, immunosuppression and long-term bacterial presence (Bandyopadhaya et al., 2012, 2016b). It appears that host tolerance training by 2-AA represses the inflammatory response via disruption of p65:CBP/p300 (active) complex and induction of p50:HDAC1 (inactive) complex. Further studies are in progress to address this regulatory mechanisms.

Consistent with our model, several studies reported that increased levels of HDAC1 and reduction of histone H3 acetylation allows persistent *Mycobacterium tuberculosis* infection (Chandran et al., 2015), and promotes long-term survival of *Anaplasma phagocytophilum* in immune cells (Rennoll-Bankert and Dumler, 2012). Interestingly, it has been also reported that NF-κB p50 promotes latency of the human immunodeficiency virus involving HDAC1 recruitment and transcriptional repression (Williams et al., 2006). Our present study describes an intimate connection between histone acetylation/deacetylation status and relevant molecular mechanisms of intracellular signaling components. This connection most likely coordinates the 2-AA-mediated host tolerance to infection.

Collectively, our results strongly indicate that 2-AA in tolerized cells acts as a switch to maintain the chromatin state from a transcriptionally active to a “silent” state, modulating the host inflammatory signaling cascade epigenetically and involving protein–protein interactions between histone acetylase/deacetylases and p50/p65 NF-κB subunits. Taken together, we conclude that 2-AA-mediated host tolerance to bacterial burden is a result of a coordinated modulation of intracellular signaling and epigenetics regulators that leads to long-lasting epigenetic alterations. This coordinated effort may contribute to the long-term bacterial presence and host resilience to bacterial burden we observed to occur *in vivo* in tolerized animals (Bandyopadhaya et al., 2012, 2016b). The data presented here provide further evidence that QS signals play an important regulatory role in modulating host intracellular signaling pathways, and uncover aspects of immunoregulation that may contribute to the HT/R promoted by the QS molecule 2-AA. This interplay between 2-AA and host can lead to a co-evolutionary arms race, where the pathogen and host are continuously selected to avoid the cost of infection and the cost of immune clearance. Given that QS is a conserved mechanism of prokaryotes, it is likely that 2-AA-like molecules that promote similar effects may exist in other pathogens. Overall, the results presented promote a better understanding of the biological mechanisms mediating mutual pathogen–host adaptation and the causes and consequences of variation in HT/R that remain extremely limited.

## Author Contributions

AB and LR conceived and designed the study. AB and LR wrote the manuscript. AB and AT performed experiments and data analysis.

## Conflict of Interest Statement

LR is the scientific founder and scientific advisory board member of Spero Therapeutics LLC (no funding was received from Spero). The other authors declare that the research was conducted in the absence of any commercial or financial relationships that could be construed as a potential conflict of interest.

## Footnotes

1 http://rsb.info.nih.gov/ij/index.html

## Supplementary Material

FIGURE S1 Human monocyte cell viability in presence of HAT inhibitor C646. MTT assay measuring viability in THP-1 cells following treatment with increasing concentrations of C646. Cells were exposed to C646 for 24 h (*n* = 3; means ± SDs).

FIGURE S2 Acetylation of CBP/p300 is modulated by 2-AA. Western blot analysis showing decreased CBP/p300 acetylation in 2-AA-tolerized mouse macrophage cells RAW264.7 cells following 2-AA stimulation (1 h). Data are representative of three independent experiments.

FIGURE S3 2-AA tolerization induces interaction between NF-κB p50 and HDAC1 and disrupts p65-CBP/p300 binding. **(a)** Co-IP assay showing increased p50/HDAC1 interaction in 2-AA-tolerized mouse macrophage RAW264.7 cells following 2-AA stimulation (1 h). **(b)** Co-IP assay confirming that 2-AA tolerization inhibits interaction of p65 with CBP and p300 in THP-1 cells. WB, Western blot. Data are representative of three independent experiments.
