# Supplementary figures and images for: NF-κBp50 and HDAC1 Interaction Is Implicated in the Host Tolerance to Infection Mediated by the Bacterial Quorum Sensing Signal 2-Aminoacetophenone
Source: Front Microbiol. 2017 Jun 30;8:1211. doi: 10.3389/fmicb.2017.01211 (PMC5492500; doi:10.3389/fmicb.2017.01211)

Supplementary Figure 1

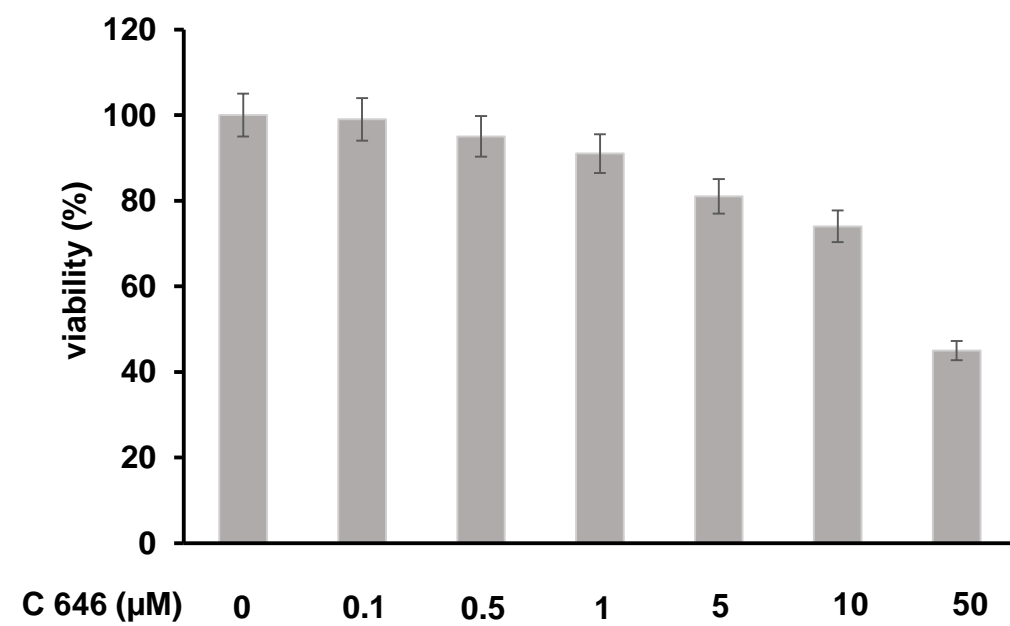

## Supplementary Figure 2

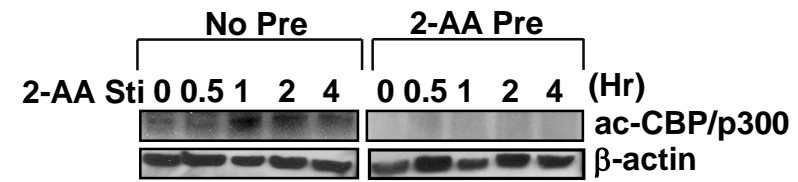

## Supplementary Figure 3

a

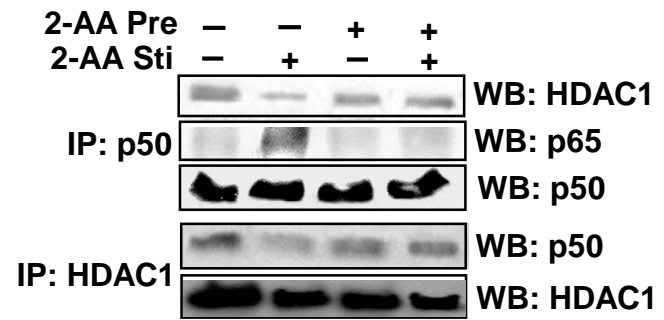

b

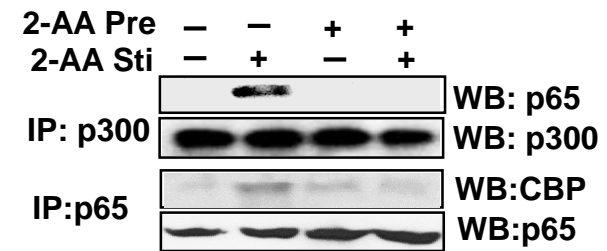

Supplement: FIGURE S1 — Human monocyte cell viability in presence of HAT inhibitor C646. MTT assay measuring viability in THP-1 cells following treatment with increasing concentrations of C646. Cells were exposed to C646 for 24 h (n = 3; means ± SDs). [file Presentation_1.PDF]
